# Supplementary material for: Innovative machine learning approach and evaluation campaign for predicting the subjective feeling of work-life balance among employees
Source: PLoS One. 2020 May 15;15(5):e0232771. doi: 10.1371/journal.pone.0232771 (PMC7228117; doi:10.1371/journal.pone.0232771)
Supplement: Attachment — Submitted filename: Innovative artificial intelligence approach and evaluation campaign for predicting the subjective feeling of work.docx [file pone.0232771.s001.docx]

**Innovative artificial intelligence approach and evaluation campaign for predicting the subjective feeling of work-life balance among employees**

This is a very interesting and innovative manuscript that merges AI technology with human feelings and work life balance/imbalance.

The authors did a great job explaining the assumptions for the project and AI utilization. I believe the paper could be accepted for publication, after following revisions:

1- Line 121 the author indicated : “We propose three components of work-family balance: time balance, involvement balance, and satisfaction balance”. I could not find the evaluation or definition of “satisfaction balance”. I recommend authors explain this factor and how they measured.
2- Line 238: authors indicated : “10 random workers were requested to fill in the study questionnaire:. How these 10 were selected? Voluntarily or selection by the investigators. What procedure were used in selection methods?
3- Lines 249-252, please rewrite and describe better. It is not comprehensive
4- Lines 253-258, please simplify and explain better for a health care or HR worker, not familiar with ANN and pell ADAM.
5- Line 368: what does the “the ratio used was 9/1” mens? please explain
6- Line 392: Please provide references for algorithm selected and explain the approach
