# Supplementary material for: Innovative machine learning approach and evaluation campaign for predicting the subjective feeling of work-life balance among employees
Source: PLoS One. 2020 May 15;15(5):e0232771. doi: 10.1371/journal.pone.0232771 (PMC7228117; doi:10.1371/journal.pone.0232771)
Supplement: Attachment — Submitted filename: Answers to the Reviewers_ANN Plos ONE-mch2.docx [file pone.0232771.s002.docx]

*Answers to the Reviewers’ Comments concerning:*

*PONE-D-20-02847
Innovative artificial intelligence approach and evaluation campaign for predicting the subjective feeling of work-life balance among employees*

Dear Reviewers and Editor,

We are very thankful for reading our paper and giving so much valuable feedback. It made us really happy that you found our work interesting. **We would like to thank you for your positive approach towards our paper.** In this revision, we have implemented all the suggested alterations and we hope that the paper can now be accepted for publication in Plos ONE.

In particular, we have reacted to the reviews and implemented the following changes:

Editor’s suggestions:

We have implemented the suggested changes wherever applicable. **The data used in this study, as suggested, has been placed in openly available repository.** We have added the missing references to tables and figures.

Reviewer #1’s suggestions:

*I want to congratulate you for your present work and interest in publication. It will be a valuable source of knowledge for interested researchers on the field.
In my professional opinion, your text is relevant and suitable but needs a few improvements to achieve the expected quality for the journal. I will go through them in order of appearance in your manuscript:*

Thank you so much for your kind, heartwarming words!

*[Abstract]
Dear authors. Please give more insights about the methodology, models and the results obtained on your abstract, this section must summarize the project as possible, Have in mind that could be the only part of your work that could be read in a quick search for external researchers.*

We have extended description of the methodology and the relevant summary for the method was added. We have also given more details on the results in the abstract. (pp. 2 -3)

*[Introduction]
Artificial intelligence is a wide knowledge field. I strongly suggest to narrow your terms and use “machine learning” or “deep learning” instead. This should be applied even to the title of the article.*

Indeed, we agree with the Reviewer – many thanks for this comment.

As suggested, we have narrowed down the term to “machine learning”. Although in the case of our study it could have been used interchangeably with the term “deep learning”, we used the former term in order to make it clearer for more readers.

*Line 57: when you mention the group of 800 employees as “exceptionally large”, which are your references for this comparison? Maybe other studies on similar cases? The sampling of the population on the determined geographical area? Please clarify.
Also, at this point will be useful for the readers to make clear about the geographical and demographic limitations of your research. Please clarify.*

Those points have been clarified in the revised version (Introduction, page 4).

*[Materials and Methods]
Line 121: Please make a further description on the three chosen variables for the experiments. As they are written “time balance”, “involvement balance” and “satisfaction balance” are quite ambiguous and hard to measure for the readers.
After reading the section is clear that there is no consensus on the definition or how to measure the WLB. So, it’s important to specify which of the exposed will be the definition used specifically for this research. Or, if you’re introducing your own definition, please make it clear.*

The three kinds of balance referred to one of the approaches found in the literature, not our own approach. We have clarified that and made the fact that we use our own definition stand out more.

*As show in the State of the art, the innovation value of your research seems to be only comparable with projects on Poland. Is the state of the art limited for the country or aren’t examples available outside it?*

We have clarified this matter by adding more information (State of the Art, pp. 10-11 as well as Introduction, p. 4).

*In the state of the art you review models based on Fuzzy Cognitive maps and ANN for similar research topics. This reinforces my idea that you should be clearer on the real innovative value of the research. Please try to describe the major differences and improvements in comparison of these projects.*

Thank you so much for pointing it out! We have rebuilt and enhanced the whole State of the Art section so that it explains in which way the other studies differed and why our study was innovative.

*Link to reference (25) is broken. Please find an alternate one.*

It has been checked and should be working now.

*Line 237: After reading the referenced paper on (5) I assume that the 16 branches of the economy are the “16 sections of the Polish Classification of Business Activities” but as a foreign reader it was difficult to me to realize that. Please make it clear on this text.*

We have made it clearer, by adding more details in the section “Dataset description”, p. 13. Thank you for pointing this out.

*[Architecture of the AI tool]
What was your criteria for selecting that architecture for the ANN?
Did you make other experiments with alternative configurations?
Even though the results were favorable, I’m concerned about the model optimization process. The architecture appears to be an arbitrary choice. That’s not necessarily wrong, but it will be better if you explain your decision.*

We have included an explanation of the choice of architecture in the text (Page 19, The section “Architecture of the Machine Learning tool” and onwards).  *[Data preprocessing and the experimental pipeline]
In order to improve the readability and structure of the manuscript, I recommend you to move the details of the feature selection techniques used on the experiments from the line 390 to 394 to this section. Please give a further description of the 10 selected features and try to justify the usage of the SelectKBest algorithm over other alternatives.*

We have moved the section to the suggested place. We have also enumerated the selected features.

*[Results]
Was your training dataset balanced? I couldn’t find anywhere the ratio of observations for each of the 4 categories. This could be decisive when calculating the accuracy of the predictions. Please clarify this or attach the classification report including other metrics like f1, precision and recall, ROC AUC, etc. I’m afraid that under this circumstances accuracy won’t be enough to answer the Research Question 1.
The selected method for*

More details of the balancing process were included in the section, along with the respective numbers of class instances before and after balancing. A classification report table was also added.

*[Conclusions]
I would like to read here your conclusions and personal appreciations about the work done using ANN models. Do you conclude it was a good approach? Could you compare with other possibilities?
Please, be specific here on the findings on which were the more meaningful variables for the prediction. This information could be extracted from the results of the feature selection algorithm.
Don’t hesitate to tell us detailed technical conclusions of your work. It may be important for the researchers to know about the troubles you had to face, your thoughts and considerations over the data extraction and preprocessing, the used model and the validation and evaluation of the results.*

Many thanks for this comment – indeed more technical details are placed now in the text. In particular, the conclusions on the use of ANN were added.

*Reviewer #2: This is a well written paper and an innovative approach. Please see my comments on the attached file. I strongly encourage the changes that i recommended be implemented before acceptance for publication.*

*This is a very interesting and innovative manuscript that merges AI technology with human feelings and work life balance/imbalance.*

*The authors did a great job explaining the assumptions for the project and AI utilization. I believe the paper could be accepted for publication, after following revisions:*

**Thank you so much for the positive opinion and your comments! We have implemented all the changes you have suggested.**

1- *Line 121 the author indicated : “We propose three components of work-family balance: time balance, involvement balance, and satisfaction balance”. I could not find the evaluation or definition of “satisfaction balance”. I recommend authors explain this factor and how they measured.*

Thank you for drawing our attention to this. „We” actually referred to the authors of the quoted article, not to us, the authors of the paper. It could have been confusing. We have clarified and modified the text, so it is unambiguous.

2- *Line 238: authors indicated : “10 random workers were requested to fill in the study questionnaire:. How these 10 were selected? Voluntarily or selection by the investigators. What procedure were used in selection methods?*

We have provided some additional information on the procedure (page 13, section Dataset description).

3- *Lines 249-252, please rewrite and describe better. It is not comprehensive*

Thank you for pointing that out. The sentence has been changed.

4- *Lines 253-258, please simplify and explain better for a health care or HR worker, not familiar with ANN and pell ADAM.*

We have added a word of explanation with regards to the ADAM optimizer and hyperparameters in general (Section: Architecture of the Machine Learning Tool, page 19).

5- *Line 368: what does the “the ratio used was 9/1” mens? please explain*

We have included further explanation of the subject matter and we hope this is now clear.

6- *Line 392: Please provide references for algorithm selected and explain the approach*

We have added a word of explanation of the algorithm, and the reference

**Once again, many thanks for the time of the reviewers, their careful reading and valuable comments. We hope that the paper can now be accepted and published, and we hope it will ‘resonate’ within the scientific community.**
